# Repeated parallel differentiation of social learning differences in benthic and limnetic threespine stickleback fish

**DOI:** 10.1098/rsbl.2023.0208

**Published:** 2023-07-26

**Authors:** Jason Keagy, Whitley Lehto, Ross Minter, Sarah Machniak, Oynx Baird, Janette W. Boughman

**Affiliations:** ^1^ Department of Ecosystem Science and Management, The Pennsylvania State University, University Park, PA 16802, USA; ^2^ Department of Integrative Biology, and; ^3^ Michigan State University, 48824, USA

**Keywords:** social learning, delayed local enhancement, stickleback, parallel evolution, reverse speciation

## Abstract

Individuals can reduce sampling costs and increase foraging efficiency by using information provided by others. One simple form of social information use is delayed local enhancement or increased interest in a location because of the past presence of others. We tested for delayed local enhancement in two ecomorphs of stickleback fish, benthic and limnetic, from three different lakes with putative independent evolutionary origins. Two of these lakes have reproductively isolated ecomorphs (species-pairs), whereas in the third, a previously intact species-pair recently collapsed into a hybrid swarm. Benthic fish in both intact species-pair lakes were more likely to exhibit delayed local enhancement despite being more solitary than limnetic fish. Their behaviour and morphology suggest their current perceived risk and past evolutionary pressure from predation did not drive this difference. In the hybrid swarm lake, we found a reversal in patterns of social information use, with limnetic-looking fish showing delayed local enhancement rather than benthic-looking fish. Together, our results strongly support parallel differentiation of social learning differences in recently evolved fish species, although hybridization can apparently erode and possibly even reverse these differences.

## 1. Introduction

Animals must gather, process and act on information about their surroundings to efficiently forage. Rather than relying solely on private information through direct sampling of the environment, animals can use information provided by other individuals (often inadvertently) to reduce sampling costs and increase foraging efficiency [1–4]. One simple form of this social learning is *local enhancement*, which is when an area becomes more attractive because of the presence and/or behaviour of other individuals [5–7]. If the focal individual visits the location after other individuals have moved on, this is called *delayed local enhancement* [5,6].

Several hypotheses have been proposed for the expected phylogenetic distribution of reliance on social information. The first and most popular hypothesis (social intelligence) is that social species evolve to take advantage of the greater amount of social information available to them and are thus expected to rely more on social information than solitary species [8,9]. A second non-mutually exclusive hypothesis (predation risk) suggests species more at risk for predation rely more on social information because the costs of using individual information are increased [5]. However, the use of past social information could trade-off against maintaining group cohesion and leaving the group might have high fitness consequences [10]. Therefore, we propose a third hypothesis: the ‘group cohesion trade-off’ hypothesis that social species rely less on certain types of social information when maintaining group cohesion is important.

An ideal system to test these hypotheses is a group of closely related species differing in social behaviour and predation risk. After the last ice age, marine threespine sticklebacks (*Gasteroseus aculeatus*) colonized multiple lakes in British Columbia (BC) and subsequently independently adapted to two distinct ecological niches, the ‘limnetic’ and ‘benthic’, in the process becoming reproductively isolated and thus separate species under the Biological Species Concept [11,12]. Limnetic fish spend most of their lives feeding together in groups (shoals) in the open-water pelagic zone whereas benthic fish tend to be more solitary and spend most of their lives foraging in the densely vegetated littoral zone [13,14]. The increased propensity of limnetic fish to shoal and their longer spines and more extensive armour plating are probable adaptations to higher predation pressure as adults compared to benthic fish [14,15], which is supported by poorer survival of trout predation in pond experiments [16].

We examined delayed local enhancement in sticklebacks from three different lakes, two with an intact, reproductively isolated pair of limnetic and benthic species (intact ‘species-pairs’, Paxton and Priest Lakes [17]) and one that historically contained a reproductively isolated species-pair that collapsed into a hybrid swarm after the introduction of crayfish (Enos Lake [18–21]). Thus, we can test for replicated divergence in social information use in limnetic-benthic species-pairs. The ‘social intelligence’ hypothesis predicts limnetic fish will use delayed local enhancement more than benthic fish. If current behavioural and morphological adaptations reflect predation pressure, then the ‘predation risk’ hypothesis also predicts limnetic fish will use delayed local enhancement more than benthic fish. To further distinguish between these hypotheses, we assessed behavioural measures related to boldness as an indicator of a fish's assessment of predation risk. The ‘group cohesion trade-off’ hypothesis makes opposite predictions to these two hypotheses; it predicts limnetic fish will use delayed local enhancement *less* than benthic fish.

## 2. Material and methods

Adult fish were wild caught in 2011 and 2012 from Enos Lake (Vancouver Island, BC) and Paxton and Priest Lakes (Texada Island, BC). Canada considers these fish to be endangered and so carefully controls collection, limiting sample sizes. Only fish that resembled limnetic or benthic fish in body shape were collected from Enos Lake [22]; we refer to them as limnetic-like and benthic-like. Fish were housed at Michigan State University at 15°C and a light : dark cycle that tracked natural changes in daylight in BC. We fed the fish defrosted brine shrimp (*Artemia* sp.) and bloodworms (*Chironomus* sp.) once per day (except where noted below).

All experiments were conducted on non-reproductive fish 4–12 months after capture in 2012 and 2013, because reproductive state can influence social information use [23]. Demonstrators were female laboratory-reared fish of the same ecotype as the observer. Fish were not fed for 24 h prior to trials to increase their motivation.

**Figure 1.**
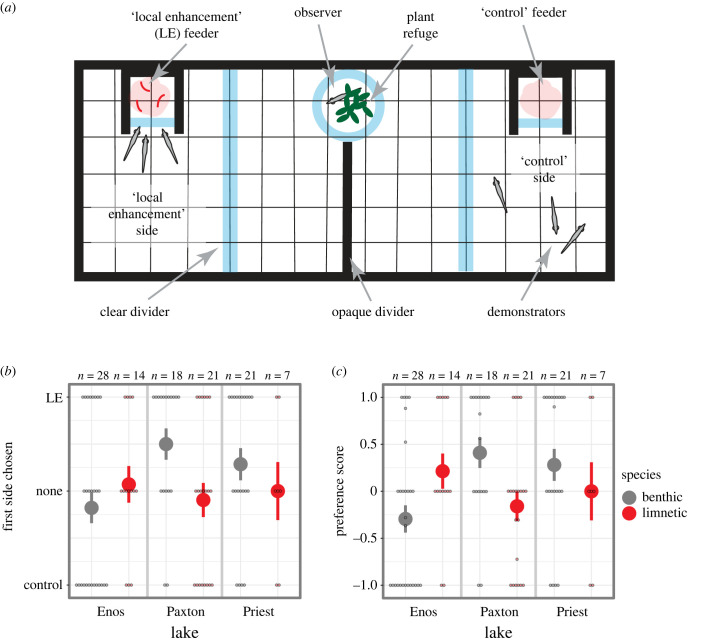
Social information use in benthic and limnetic sticklebacks. (*a*) Diagram of experimental set-up during a feeding. (*b*) Benthic fish in both intact species-pair lakes (Paxton and Priest Lakes) chose the local enhancement side first more often than limnetic fish, but this pattern was reversed in Enos Lake. (*c*) Similar patterns are seen using a preference score that takes into account time spent on either side. Small circles are raw data points. Large circles and vertical lines are mean ± s.e.

Our experimental procedure was based on those used previously with sticklebacks [5,7]. The experimental tank (110 l with a 76 × 31 cm footprint and 43 cm water depth, figure 1*a*) was divided into three equal-sized sections using clear acrylic dividers. The sections on the right and left housed demonstrator shoals (three fish each), which could swim freely. The middle section housed the observer. The observer was placed in a clear cylinder (10.5 cm diameter, 14.2 cm height) with an artificial plant refuge. An opaque divider (white corrugated plastic) was placed in the centre of the middle compartment to limit interaction between demonstrator shoals. The interior of the tank, including the floor, was lined with white corrugated plastic to eliminate reflections on the tank walls and prevent fish from seeing outside the tank. Feeders were placed in the centre of each demonstrator section against the front wall. The feeders were 5 × 5 × 53.5 cm high columns with opaque sides and a transparent front, such that the demonstrators, but not the observer, could see the contents. A Canon VIXIA M40 HD camcorder mounted above connected to a monitor allowed live observation and video-recording.

Each observer was tested once. To reduce potential stress during the experiment (e.g. owing to neophobia), the observer was first allowed to explore for approximately 40 min a tank identical to the experimental tank, but without dividers. At the start of each trial, two demonstrator shoals and the observer were placed in their compartments and given 10 min to acclimate. A 1 ml transfer pipette was then used to feed each feeder every 90 s for 10 min (six times total). Patch status was randomly assigned in a balanced way; with the ‘local enhancement’ side fed three bloodworms suspended in water and the ‘control’ side fed just water in which the bloodworms had been defrosted (figure 1*a*). Demonstrators pecked at the transparent feeder front as the bloodworms sunk to the bottom of the column, where they could be eaten through a 2.5 cm tall slot. This design prolonged demonstration, making it a salient cue for the observer.

One minute after the final feeding, the observer was visually isolated and demonstrator shoals were removed. The feeders were removed, quickly cleaned and replaced. All dividers and the cylinder were then removed. Using JWatcher 1.0, we recorded the observer's latency to move and all transits into the centre, left and right sides for the next 5 min. We also recorded time spent in cover of the artificial plant (defined as any part of their body covered by the plant). If there was no movement for 10 min after release, the trial was ended.

One hundred and thirty-one total trials were run; however, there were two mis-trials (experimenter error with trial set-up) and 20 trials in which the observer never moved once released, leaving 109 trials. Consistent with previous studies (e.g. [5]), we quantified behaviour for the first 90 s after the observer started moving.

Here we focus our analyses of fish choice behaviour on those fish that left the centre region (*n* = 73 fish). When we instead analysed the complete dataset, results were similar (see the electronic supplementary material). We analysed the proportion that first chose the local enhancement side with a binomial logistic regression model (using the *glm* function with the ‘logit’ link function in the *stats* package [24]). We calculated a preference score as (time on local enhancement side – time on control side)/(total time on either side). We analysed preference scores with a left (all time on the control side = −1) and right censored (all time on the local enhancement side = + 1) tobit regression model (using the *tobit* function in the *AER* package [25]). We analysed latency to move and time hiding in the plant data for the complete dataset with a linear model (using the *lm* function in the *stats* package [24]) after a square-root transformation to improve normality of residuals. Our models for intact species-pair lakes had fixed effects of lake (Paxton and Priest), species (limnetic or benthic) and their interaction. Our models contrasting differences between intact species-pair lakes versus Enos Lake where species have collapsed had fixed effects of intact (yes = Paxton and Priest Lakes combined, or no = Enos Lake), species (limnetic/limnetic-like or benthic/benthic-like) and their interaction. Analysis of deviance or ANOVA tables were generated using the *Anova* function from the *car* package [26]. All statistical analyses were done in R v. 4.0.2 [24]. Figures were drawn using the *ggplot2* package [27].

## 3. Results

### (a) Are there species differences in use of social information?

#### (i) First choice

We first asked whether species in both lakes with intact species-pairs (Paxton and Priest) differed in their propensity to first choose the local enhancement side. Benthic fish were more likely to first choose the local enhancement side than limnetic fish (*χ*^2^_1_= 5.41, *p* = 0.02; figure 1*b*). There was no significant lake × species interaction (*χ*^2^_1_= 0.52, *p* = 0.47), supporting parallel species differences across lakes. There was also no main effect of lake (*χ*^2^_1_= 0.23, *p* = 0.63).

Next, we asked how limnetic and benthic fish from intact species-pair lakes compared to limnetic-like and benthic-like fish from Enos Lake. We found a significant intact × species interaction (*χ*^2^_1_= 4.21, *p* = 0.04), caused by limnetic-like and benthic-like fish from Enos Lake showing the opposite pattern as limnetic and benthic fish from intact species-pair lakes (figure 1*b*); in Enos Lake, limnetic-like fish tended to choose the local enhancement side more than benthic-like fish.

#### (ii) Preference score

We also calculated a preference score, which could be more nuanced than a fish's first choice. However, fish generally stayed in the region they initially chose (the first choice and preference scores were highly correlated: *R* = 0.89, *t*_71_ = 16.58, *p* < 2.2^−16^). Indeed, the results of analyses using the preference score were parallel to those examining the initial choices made (figure 1*c*; tables 1 and 2).

**Table 1.** Preference score, intact species-pair lakes (Paxton and Priest Lakes). (Italics indicate statistically significant effect, *p* < 0.05.)

effect	*χ*^2^	d.f.	*p*
lake	0.002	1	0.97
*species*	*4.05*	*1*	*0.04*
lake × species	0.17	1	0.68

**Table 2.** Preference score, intact species-pairs lakes compared to Enos Lake. (Italics indicate statistically significant effect, *p* < 0.05.)

effect	*χ*^2^	d.f.	*p*
intact	3.75	1	0.05
species	0.22	1	0.64
*intact × species*	*8.87*	*1*	*0.003*

### (b) Are there species differences in latency to move and hiding?

#### (i) Latency to move

**Figure 2.**
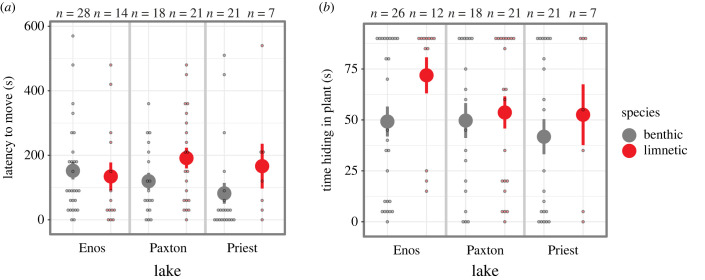
Traits related to risk aversion in benthic and limnetic sticklebacks. (*a*) In both lakes with intact species-pairs, benthic fish moved sooner than limnetic fish. This pattern was reversed in Enos Lake. (*b*) Benthic(-like) fish did not vary from limnetic(-like) fish in their time spent hiding in the plant. Small circles are raw data points. Large circles and vertical lines are mean ± s.e. Raw data shown here; analyses were done on square-root-transformed data (see Material and Methods).

When we considered fish from both intact species-pair lakes (Paxton and Priest), we found a significant effect of species (*F*_1, 63_ = 5.33, *p* = 0.024; figure 2*a*), with limnetic fish taking longer to move once released compared to benthic fish. There was no significant lake × species interaction (*F*_1,63_ = 0.21, *p* = 0.65), supporting parallel species differences across lakes. There was also no significant lake effect (*F*_1,63_ = 2.70, *p* = 0.11).

When we compared fish from intact species-pair lakes to those in Enos Lake, we found a significant intact × species interaction (*F*_1,105_ = 5.45, *p* = 0.022), caused by limnetic-like and benthic-like fish from Enos Lake showing the opposite pattern as limnetic and benthic fish from intact species-pair lakes (figure 2*a*); in Enos Lake, limnetic-like fish tended to move sooner than benthic-like fish.

#### (ii) Time hiding

We found no differences between species in intact species-pair lakes in the time fish spent hiding under the plant (*F*_1,63_ = 0.56, *p* = 0.45; figure 2*b*), and no significant interaction between species and lake (*F*_1,63_ = 0.09, *p* = 0.77) or main effect of lake (*F*_1,63_ = 0.68, *p* = 0.41). There was also no difference between fish from intact species-pair lakes from those in Enos Lake (figure 2*b*; electronic supplementary material, table S1).

## 4. Discussion

We tested individual threespine stickleback fish on the ability to use the feeding behaviour of others to locate a food patch. In two lakes with reproductively isolated species adapted to different ecological niches, we found strong evidence for parallel evolution of species differences in social information use; benthic fish used past social information to locate a food patch whereas limnetic fish did not. These differences were not maintained and seemingly reversed in Enos Lake, where the two former species have been hybridizing after anthropogenic disturbance. Given that benthic fish from intact species-pair lakes used the social information whereas the limnetic fish did not, we can reject the hypothesis that sociality selected for increased reliance on this type of social information. In addition, despite using past social information more, benthic fish did not behave as if they were more risk averse than limnetic fish. In fact, limnetic fish took longer to move, suggesting risk aversion, at least when alone, as might be expected given other morphological and behavioural traits suggesting more intense predation pressure [14,15]. This allows us to reject the hypothesis that predation risk selected for use of delayed local enhancement information. Instead, our data suggest that limnetic fish from intact species-pair lakes rely on past social information less, supporting the ‘group cohesion trade-off’ hypothesis. Indeed, Odling-Smee *et al*. [28] found that food-limited limnetic fish from Paxton and Priest Lakes showed a preference for a shoal over food when offered each on opposite sides of a tank, suggesting that limnetic fish find being in a shoal especially important.

It was somewhat surprising that many individuals (such as limnetic fish from intact species-pair lakes) did not show delayed local enhancement. Two independent studies previously demonstrated delayed local enhancement with UK stream-collected threespine sticklebacks [5,7]. However, these fish were unable to do the more complicated cognitive task of using social cues to determine food patch quality differences more subtle than presence versus absence (public information) [5,7]. Indeed, previous data from a number of different populations, including Paxton limnetic and benthic populations, suggest that unlike ninespine sticklebacks (*Pungitius pungitius*), threespine sticklebacks do not use public information [29,30].

Given previous findings of parallel evolution of better spatial learning in benthic fish compared to limnetic fish [28,31], it is possible that benthic fish are better at learning in general. Benthic sticklebacks from Paxton and Priest Lakes also have larger relative brain volumes than limnetic fish from these lakes [32], although brain size is a very imperfect proxy for cognitive ability [33]. Interestingly, these brain size differences are reversed in Enos Lake [32], similar to our current findings regarding delayed local enhancement. Sticklebacks are a model system for evolutionary biology; further study of their cognition will provide an opportunity to address long-standing questions regarding the evolution of cognition and the brain.

Social interaction has been suggested to drive the evolution of intelligence and brain size [8,9], with supporting evidence coming from a comparative primate study associating group size with brain size [8]. However, this relationship is often not supported, leading to calls for more nuanced investigation of the relationship between sociality and social intelligence (e.g. birds [34], hyenas [35]). Indeed, rarely are actual cognitive abilities assessed in tests of the ‘social intelligence’ hypothesis (but see [36–38]). Given our results, we encourage testing whether various types of social learning are associated with sociality in a larger range of animal species, preferably comparing species with recent shifts in sociality. This approach will allow a more holistic test of the ‘social intelligence’ hypothesis.

## Data Availability

Data and analysis code are available from the Dryad Digital Repository: https://doi.org/10.5061/dryad.8931zcrwb [39]. See also the electronic supplementary material [40].
